# BODIPY-cholesterol can be reliably used to monitor cholesterol efflux from capacitating mammalian spermatozoa

**DOI:** 10.1038/s41598-019-45831-7

**Published:** 2019-07-08

**Authors:** N. C. Bernecic, M. Zhang, B. M. Gadella, J. F. H. M. Brouwers, J. W. A. Jansen, G. J. A. Arkesteijn, S. P. de Graaf, T. Leahy

**Affiliations:** 1 School of Life and Environmental Sciences, Faculty of Science, The University of Sydney, Sydney, New South Wales Australia; 2 Department of Biochemistry and Cell Biology, Faculty of Veterinary Medicine, Utrecht University, Utrecht, The Netherlands; 3 Department of Farm Animal Health, Faculty of Veterinary Medicine, Utrecht University, Utrecht, The Netherlands

**Keywords:** Membrane trafficking, Assay systems, Membrane lipids

## Abstract

Capacitation is the final maturation step spermatozoa undergo prior to fertilisation. The efflux of cholesterol from the sperm membrane to the extracellular environment is a crucial step during capacitation but current methods to quantify this process are suboptimal. In this study, we validate the use of a BODIPY-cholesterol assay to quantify cholesterol efflux from spermatozoa during *in vitro* capacitation, using the boar as a model species. The novel flow cytometric BODIPY-cholesterol assay was validated with endogenous cholesterol loss as measured by mass spectrometry and compared to filipin labelling. Following exposure to a range of conditions, the BODIPY-cholesterol assay was able to detect and quantify cholesterol efflux akin to that measured with mass spectrometry. The ability to counterstain for viability is a unique feature of this assay that allowed us to highlight the importance of isolating viable cells only for a reliable measure of cholesterol efflux. Finally, the BODIPY-cholesterol assay proved to be the superior method to quantify cholesterol efflux relative to filipin labelling, though filipin remains useful for assessing cholesterol redistribution. Taken together, the BODIPY-cholesterol assay is a simple, inexpensive and reliable flow cytometric method for the measurement of cholesterol efflux from spermatozoa during *in vitro* capacitation.

## Introduction

To acquire the capacity to fertilise, spermatozoa must spend a period of time residing in the female reproductive tract undergoing the process of capacitation^1,2^. In what can be considered as the final stage of maturation, spermatozoa are subjected to series of physico-chemical transformations during capacitation that will collectively permit interaction with the oocyte and eventual fertilisation. From the time of this discovery by both Chang^1^ and Austin^2^, a vast amount of research has been dedicated to understanding capacitation and identifying the numerous processes that occur during this window of time. One of the processes of particular interest is the reverse transport of cholesterol from the sperm membrane to the extracellular environment, also known as cholesterol efflux.

Cholesterol efflux has been well documented in somatic cells as a means to regulate cellular cholesterol homeostasis^3,4^ but the pathway that exports cholesterol from the sperm membrane during capacitation in spermatozoa is not well defined^5^. Although, it can be speculated based on the current evidence of this process in spermatozoa what is required to support cholesterol efflux and how it functions in these cells. When spermatozoa are exposed to an elevation of bicarbonate they activate adenylate cyclase activity and the enhanced cAMP levels in turn activate a protein kinase A dependent signalling pathway^6^. These changes in the plasma membrane induce a concomitant increase in fluidity and decrease in stability which is paramount to facilitate cholesterol redistribution and efflux^7^. For the removal of cholesterol, it is likely that similar to somatic cells, this molecule is shuttled by transmembrane proteins like ATP-binding cassette (ABC) transporters that have been identified in spermatozoa^8,9^. Suitable cholesterol acceptors in the extracellular environment, such as serum albumin or high-density lipoproteins^10–15^ can then uptake and remove cholesterol from spermatozoa, further contributing to modifications in the plasma membrane during capacitation.

Unlike somatic cells, the function of cholesterol efflux is not for the regulation of cholesterol homeostasis nor is it likely to be a bi-directional process. In fact, several studies have provided strong evidence that cholesterol efflux is important for events leading to successful fertilisation. In both human and mouse spermatozoa, cholesterol loss from the plasma membrane has been associated with changes in tyrosine phosphorylation of sperm proteins, a post-translational modification that can manipulate sperm function to support fertilisation-related events^13,14,16^. Furthermore, the redistribution of lipid rafts in the membrane that harbour proteins involved in fertilisation has been shown to be dependent on the loss of cholesterol during capacitation^17–19^. Finally, cholesterol efflux was able to facilitate penetration of the zona pellucida and support fertilisation in several species^14,20,21^.

Since cholesterol efflux plays an integral role in the fertilising ability of spermatozoa, it is not unexpected that there is a growing interest in understanding the mechanisms involved and identifying the necessary requirements for this process. Currently, there are several methods used to quantify cholesterol efflux during capacitation. These include the use of radioactively labelled cholesterol^12,15^, thin layer chromatography^13,14^, cholesterol quantification kits like Amplex® Red^22^, filipin labelling^23–26^ or advanced techniques like the analysis of endogenous lipids with mass spectrometry^7^. Unfortunately, the issue with many of these methods is that they can be difficult to perform, can produce subjective results, measurements are based on a whole sperm population that may include deteriorated cells or they require equipment that is not easily accessible in a standard andrology laboratory. In this case, there is a strong need for a simple and reliable assay that can quantify cholesterol efflux in living sperm cells using equipment that is commonplace for assessing sperm function in single cells, such as a flow cytometer^27^.

Several fluorescent cholesterol analogues have been developed to investigate the cellular movement of cholesterol in somatic cells, particularly for emerging research investigating atherosclerosis and links to heart disease. One of these analogues that have proven to be most useful is cholesterol tagged with a boron dipyrromethene difluoride (BODIPY) fluorophore, also known as BODIPY-cholesterol (see Supplementary Fig. S1). BODIPY-cholesterol is a favourable analogue for studying cholesterol because it does not cause perturbations in model membranes and it is able to mimic the ordering properties of native cholesterol^28^. This cholesterol analogue is also extremely photo-stable and it is conveniently excited with an argon laser (488 nm) similar to other frequently used fluorophores to assess sperm function, like fluorescein isthiocyanate labelled peanut agglutinin (FITC-PNA). In addition, in somatic cells it has been shown to be a safer, more efficient and sensitive method of cholesterol efflux quantification than the traditional method of utilising radioactively labelled cholesterol^29^. Despite the fact that this analogue has only been used in somatic cells to date, the optimal properties of BODIPY-cholesterol make it an excellent candidate to use as a means to quantify cholesterol efflux from spermatozoa.

In this study, we present the development of a BODIPY-cholesterol assay that can efficiently measure cholesterol efflux from spermatozoa via flow cytometry and be readily applied to vitro capacitation research. The boar was selected as a model as successful *in vitro* capacitation protocols have been established for this species and this assay could be easily applied to these^6,7,23,30^. To ensure the reliability of the assay, the objectives of the current study were to validate the results of the BODIPY-cholesterol assay in boar spermatozoa with endogenous cholesterol loss measured on lipid extracts of incubated and washed sperm suspension by mass spectrometry. The efficacy of this assay was also evaluated against filipin labelling, a current method of assessing cholesterol redistribution and efflux.

## Results

### The BODIPY-cholesterol assay can detect and quantify cholesterol efflux in capacitating conditions

**Figure 1:**
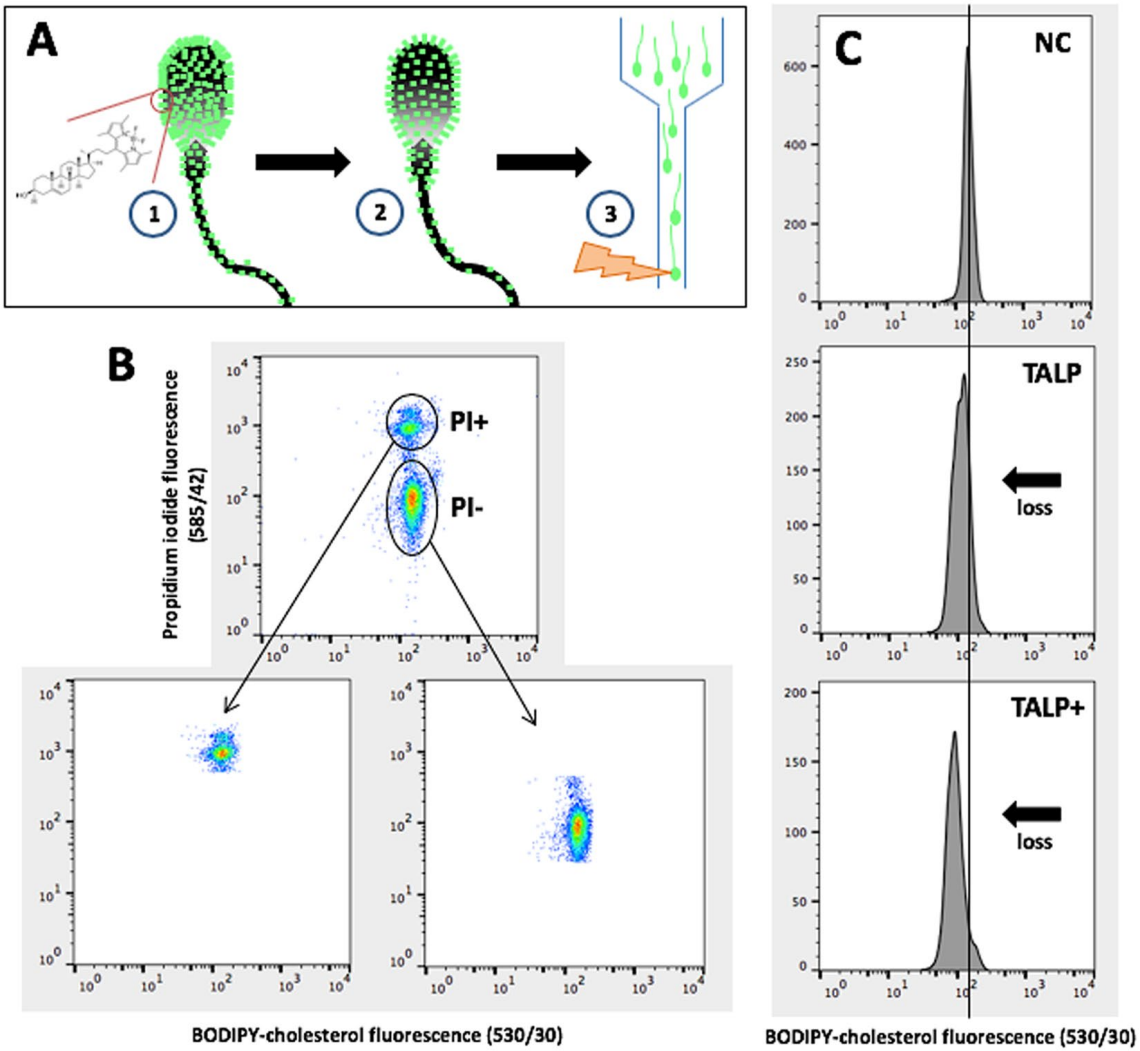
Preparation and flow cytometric analysis of BODIPY-cholesterol labelled boar spermatozoa. (**A**) To prepare spermatozoa for the BODIPY-cholesterol assay, cells are first labelled with BODIPY-cholesterol in a non-capacitating media (1), then excess label is removed via density gradient centrifugation and spermatozoa can be incubated in various capacitating conditions (2). Following capacitation (2 h), spermatozoa can be analysed with flow cytometry and to determine cholesterol efflux, the percent loss in BODIPY-cholesterol fluorescence relative to the non-capacitating control (NC) was calculated (i.e. BODIPY-cholesterol fluorescence in TALP ÷ BODIPY-cholesterol fluorescence in NC × 100). (**B**) The resulting density plot of recorded sperm events with BODIPY-cholesterol and counterstained with propidium iodide (PI) to isolate a viable (PI−) and non-viable population (PI+) for analysis. (**C**) After selecting the viable population only, a loss in BODIPY-cholesterol can be observed following 2 h incubation in capacitating conditions (as indicated by black arrow), TALP and TALP supplemented with cAMP up-regulators (TALP+) when compared to the non-capacitating control (NC).

**Figure 2:**
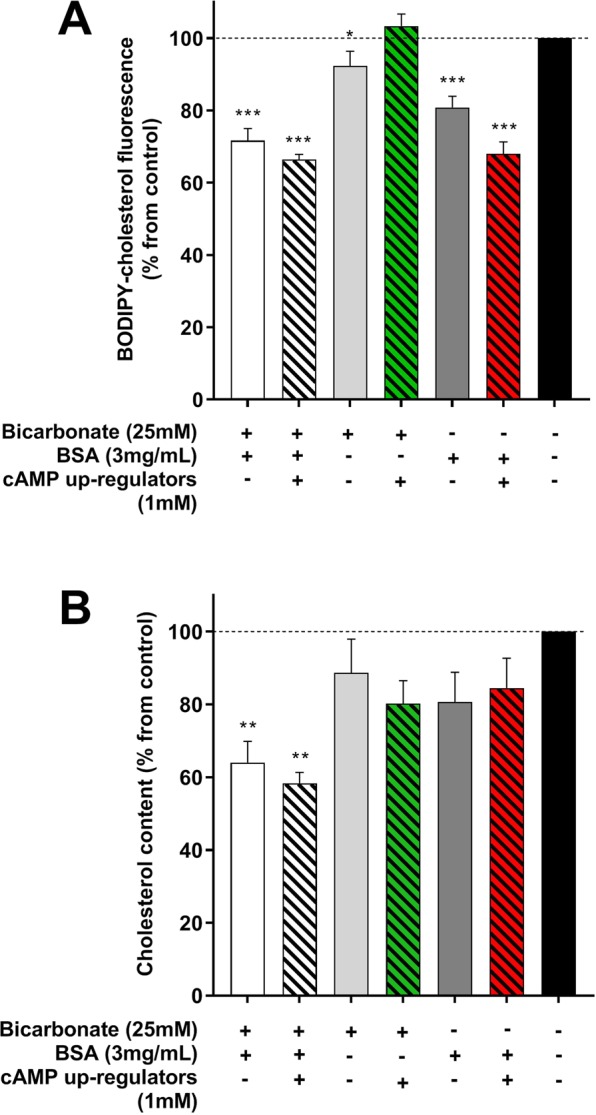
The percent loss in BODIPY-cholesterol fluorescence (**A**) and endogenous cholesterol (**B**) from boar spermatozoa relative to the non-capacitating control. Boar spermatozoa were either labelled with BODIPY-cholesterol prior to exposure to various conditions for 2 h and the efflux of BODIPY-cholesterol was tracked with flow cytometry or alternatively, sperm lipids were extracted following exposure to various conditions and subsequently analysed for endogenous cholesterol loss with mass spectrometry. (**A**) BODIPY-cholesterol efflux was highest in spermatozoa exposed to TALP supplemented with or without cAMP up-regulators (white and white hatched bar, respectively) or media without bicarbonate but supplemented with cAMP up-regulators (dark gray hatched bar). (**B**) In contrast, only TALP and TALP with cAMP up-regulators were able to support a significant loss in endogenous cholesterol. When spermatozoa were exposed to TALP devoid of BSA or bicarbonate and supplemented with cAMP up-regulators, there was a significant difference in cholesterol loss quantified by the two methods. Endogenous cholesterol loss was greater in TALP devoid of BSA with cAMP up-regulators compared with BODIPY-cholesterol ((**A** and **B**) green hatched bar) and the opposite was observed for TALP devoid of bicarbonate supplemented with cAMP up-regulators ((**A** and **B**) red hatched bar). Results are based on five (endogenous cholesterol) six (BODIPY-cholesterol) independent samples and presented as the mean percent BODIPY-cholesterol or endogenous cholesterol remaining ± SEM. *P < 0.05, **P < 0.01 and ***P < 0.0001 indicate differences from the non-capacitating control within a quantification method.

Spermatozoa were labelled with BODIPY-cholesterol and exposed to various capacitating or non-capacitating conditions. Loss of BODIPY-cholesterol from the spermatozoon was quantified using flow cytometry (Fig. 1) and cholesterol efflux was determined as the percentage change in BODIPY-cholesterol fluorescence compared to the non-capacitating control (100%) at 2 h of incubation. Spermatozoa incubated in traditional capacitation media, TALP, presented with a 28% loss of BODIPY-cholesterol (Fig. 2A; 71.7 ± 3.3% BODIPY-cholesterol remaining relative to non-capacitating control (100%); P < 0.001). Despite the upregulation of cyclic AMP using db-cAMP, caffeine and theophylline (cAMP up-regulators) supplemented to TALP media either with or without bicarbonate, these conditions induced a similar loss in BODIPY-cholesterol compared to TALP alone (66.4 ± 1.5% and 68.0 ± 3.3% BODIPY-cholesterol remaining, respectively; P > 0.05). An unexpected result observed was an apparent loss of BODIPY-cholesterol from spermatozoa exposed to TALP without bicarbonate or BSA (Fig. 2A; 80.8 ± 3.1% and 92.3 ± 4.1%, respectively). Both of these media are regarded as non-capacitating owing to the removal of vital components, bicarbonate or BSA, yet a decline in BODIPY-cholesterol was observed. In spite of this observation, the loss of BODIPY-cholesterol from spermatozoa incubated in either of these conditions was found to be significantly less than that measured in cells exposed to TALP with or without cAMP up-regulators and TALP without bicarbonate supplemented with cAMP up-regulators (P < 0.05). To verify that cholesterol efflux measured by BODIPY-cholesterol occurred as a result of capacitation, tyrosine phosphorylation was assessed by both Western blotting and immunofluorescence (Supplementary Figs S2 and 3). As expected, conditions with extensive tyrosine phosphorylation corresponded with the presence of significant cholesterol efflux. In all *in vitro* conditions, sperm viability was maintained above 50%. The exception was for spermatozoa incubated in TALP supplemented with cAMP up-regulators either with or without bicarbonate, whereby viability was at its lowest when compared to the non-capacitating control (41.7 ± 2.8%, 42.1 ± 1.9% and 81.9 ± 1.9% (control), respectively; P < 0.001).

**Figure 3:**
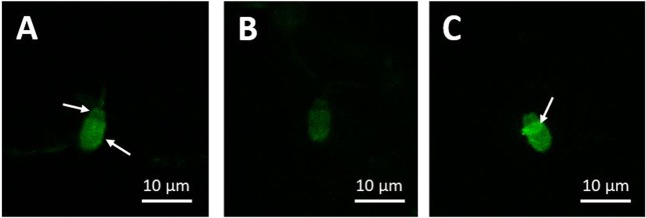
Representative images of viable (**A** and **B**) and non-viable (**C**) BODIPY-cholesterol labelled boar spermatozoa following 2 h in non-capacitating and capacitating conditions. Note that labelling is higher in the apical sperm head than that in the post-equatorial region (indicated with arrows), which is most obvious in sperm that have not undergone capacitation (**A**). Upon incubation with capacitating conditions, BODIPY-cholesterol fluorescence reduces across the whole sperm head (**B**), which visually demonstrates the loss of this cholesterol analogue from the plasma membrane during capacitation. In non-viable spermatozoa, as assessed with propidium iodide (PI), BODIPY-cholesterol fluorescence was more intense across the entire sperm head, particularly in the equatorial region (**C**; indicated with an arrow), demonstrating potential intracellular labelling. Scale bar = 10 µm.

When boar spermatozoa were examined with fluorescent microscopy, a consistent pattern of labelling was observed on the sperm head, with the apical region presenting higher fluorescence than that in the pre-equatorial region (Fig. 3). Following exposure to capacitating conditions (i.e. TALP), fluorescence across the whole sperm head was noticeably reduced compared to the non-capacitating control, indicating the loss of BODIPY-cholesterol from the plasma membrane (Fig. 3A and B). These imaging results verify what was detected with flow cytometry in non-capacitating and capacitating conditions. As such, it is recommended that flow cytometry is used to assess changes in BODIPY-cholesterol fluorescence in order to gain a reliable and quantifiable measure of cholesterol efflux.

### The cholesterol analogue, BODIPY-cholesterol, displays similar efflux properties to that of endogenous sperm cholesterol

The mass spectrometric (MS) analysis of lipids in spermatozoa exposed to TALP for 2 h either with or without cAMP up-regulators, quantified up to a 41% loss of endogenous cholesterol under these conditions (Fig. 2B; 64.0 ± 5.8% and 58.2 ± 3.1% endogenous cholesterol remaining relative to the non-capacitating control (100%), respectively; P < 0.01). These conditions also corresponded with the presence of extensive tyrosine phosphorylation, indicating that capacitation was supported (Supplementary Figs 2 and 3). This significant loss in cholesterol was not observed in spermatozoa exposed to conditions devoid of BSA or bicarbonate, even with cAMP up-regulators (Fig. 2B; P > 0.05). When the MS analysis results were compared with that measured by the BODIPY-cholesterol assay, there were very few significant differences between the two methods of quantifying cholesterol efflux (Fig. 2A and B). For spermatozoa exposed to TALP either with or without cAMP up-regulators, cholesterol efflux measured by BODIPY-cholesterol was validated with MS analysis (P > 0.05), strongly indicating that under optimal conditions for capacitation, cholesterol efflux can be reliably quantified with BODIPY-cholesterol. There were only two conditions whereby a greater loss of cholesterol was measured by one method compared to the other, these being TALP devoid of BSA or bicarbonate and supplemented with cAMP up-regulators (Fig. 2A and B). Interestingly, it was the BODIPY-cholesterol assay that appeared to quantify greater cholesterol efflux in TALP devoid of bicarbonate with cAMP upregulation, which is a condition expected to support this process (BODIPY-cholesterol: 68 ± 3.3% remaining; MS analysis: 84.5 ± 8.2% remaining; P < 0.05). In contrast, there was no evidence of cholesterol loss as measured by BODIPY-cholesterol in media devoid of BSA even with cAMP upregulation, a result that is also expected as a cholesterol acceptor is thought to be mandatory for the efflux of cholesterol to the extracellular environment (BODIPY-cholesterol: 103.4 ± 3.3% remaining; MS analysis: 80.2 ± 6.3% remaining; P < 0.05).

### Isolation of the viable population provides the most reliable method for quantifying cholesterol efflux with the BODIPY-cholesterol assay

**Figure 4:**
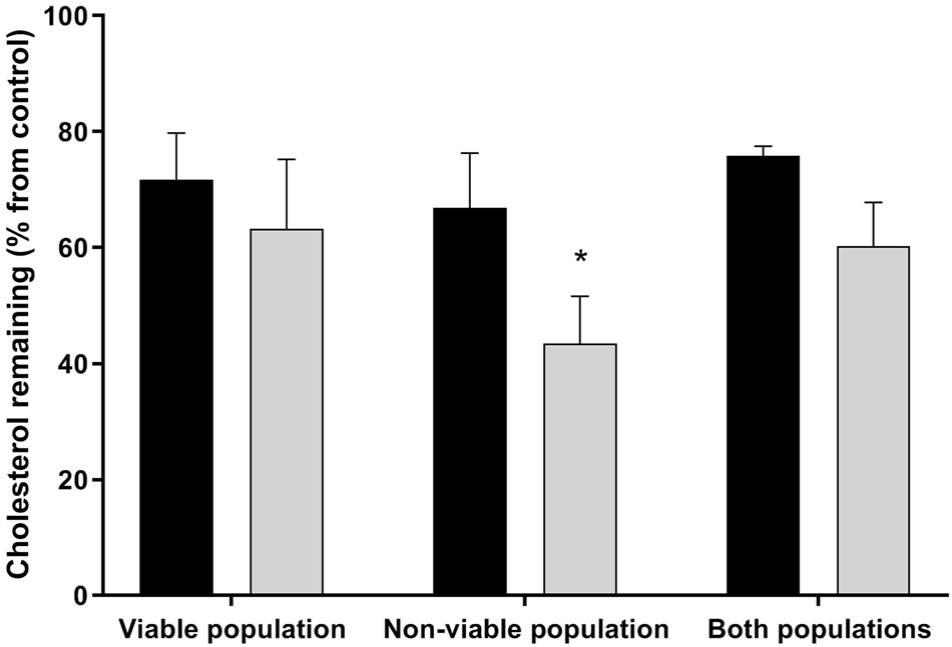
The percentage loss of cholesterol from boar spermatozoa exposed to capacitating conditions as measured by the BODIPY-cholesterol assay (black bars) and mass spectrometric (MS) analysis (gray bars) for populations segregated based on viability. To isolate sperm populations, spermatozoa were stained with propidium iodide (PI) and either BODIPY-cholesterol labelled spermatozoa were gated for these different populations or cells were sorted based on these populations and then sperm lipids were analysed with mass spectrometry. Only in non-viable cells was there a major discrepancy between cholesterol efflux measured by BODIPY-cholesterol and lipid analysis. Results are based on six independent samples for both measures and presented as the mean percent cholesterol remaining ± SEM. *P < 0.05 indicate differences between cholesterol efflux measured by BODIPY-cholesterol and MS analysis for each sperm population.

As the BODIPY-cholesterol assay is flow-cytometry based, it is possible to discriminate the viable cell population and assess cholesterol efflux from this population only. Since mass spectrometric (MS) analysis of endogenous cholesterol uses the whole sperm population, we were interested in examining cholesterol efflux across different populations segregated based on cell viability for both methods. To segregate these populations for MS analysis, spermatozoa were sorted for viability using propidium iodide (PI) with a flow cytometric cell sorter and then sperm lipids were extracted for the assessment of cholesterol efflux (see Supplementary Fig. S4). Cholesterol efflux quantified by BODIPY-cholesterol in viable cells was similar to the loss of endogenous cholesterol measured with MS analysis (Fig. 4; 71.7 ± 3.3% and 63.2 ± 12% remaining relative to non-capacitating control (100%), respectively; P > 0.05). However, the opposite was observed for the non-viable sperm population, whereby endogenous cholesterol loss exceeded that measured by BODIPY-cholesterol (43.5 ± 8.1% and 66.9 ± 3.8% remaining, respectively; P < 0.05). When the population consisted of both viable and non-viable cells, like the viable population, there was little disparity between the two methods of cholesterol efflux quantification (Fig. 4; P > 0.05). Although it is worth noting that the difference between BODIPY-cholesterol and MS analysis was almost double what was observed in the viable population alone (15.6% vs 8.5%, respectively). Viability remained relatively consistent between sorted spermatozoa destined for MS analysis and those labelled with BODIPY-cholesterol across both media conditions (TALP: 56.7 ± 1.8% vs 61.5 ± 3.3%; non-capacitating control: 89.6 ± 2.7% vs 81.9 ± 1.9% for MS analysis and BODIPY-cholesterol respectively)

### BODIPY-cholesterol is a superior method for measuring cholesterol efflux but filipin can be effectively used to detect cholesterol redistribution

**Figure 5:**
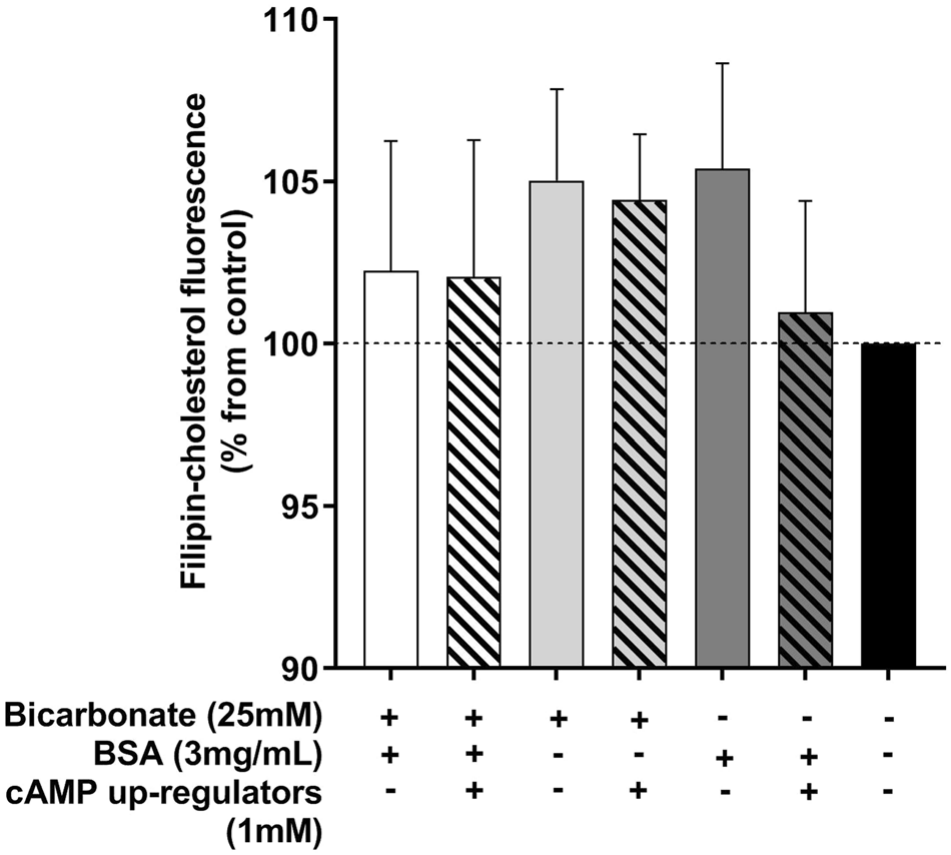
The percentage change in filipin fluorescence measured from boar spermatozoa incubated in various capacitating and non-capacitating conditions. Following fixation and labelling with filipin complex, filipin fluorescence was quantified with flow cytometry. There was no detectable difference in the percentage change in fluorescence across any of the conditions when compared to the non-capacitating control (black bar). Results are based on six independent samples and presented as the mean percent in filipin-cholesterol compared to the non-capacitating control ± SEMs.

To quantify cholesterol efflux and observe patterns of cholesterol redistribution, spermatozoa were labelled with a filipin complex (≥70% filipin III) and the fluorescence measured with flow cytometry or visualised with fluorescent microscopy, respectively. When filipin was measured with flow cytometry, there was surprisingly no detectable difference in the fluorescence of labelled spermatozoa after exposure to capacitating or non-capacitating conditions (Fig. 5; P > 0.05). If these results were compared against those measured with the BODIPY-cholesterol assay, it is evident that BODIPY-cholesterol is not only able to detect cholesterol efflux but can quantify the capacity for loss in spermatozoa unlike filipin (Fig. 2A).

**Figure 6:**
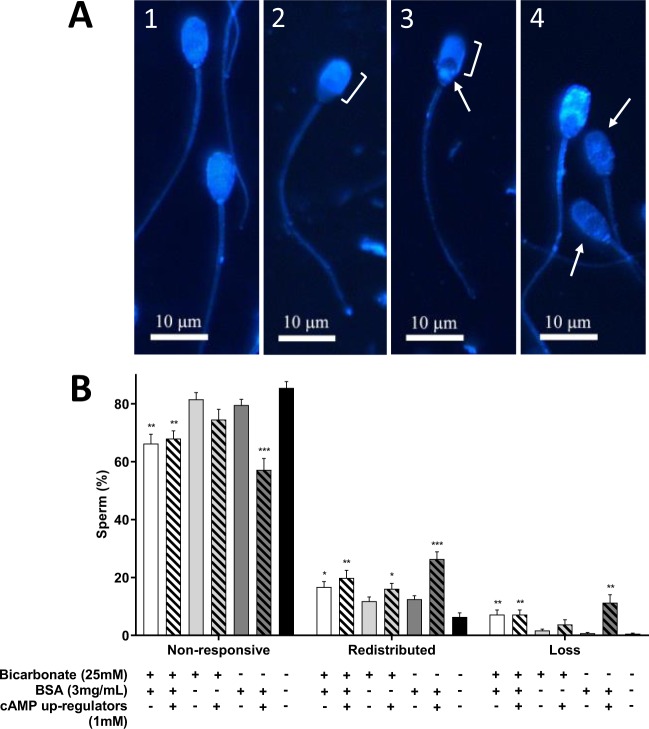
Predominant patterns of fluorescent filipin-cholesterol labelling and the frequency of each of these patterns observed on fixed boar spermatozoa following incubation in capacitating and non-capacitating conditions for 2 h. (**A**) Uniform filipin labelling was observed across the entire sperm head in non-capacitating cells (1). In capacitating cells, filipin-cholesterol complexes concentrate in the apical and pre-equatorial region of the sperm head (indicated by bracket; 2). As in (2) but there were filipin-cholesterol complexes residing in the post-equatorial region of the sperm head (post-equatorial region indicated by an arrow; 3). Cells that exhibited a loss of filipin-cholesterol complexes were identified by the lower fluorescence when compared to non-responsive cells or those with cholesterol redistribution (indicated by arrows; 4). Scale bar = 10 µm. (**B**) Spermatozoa exposed to TALP supplemented with or without cAMP up-regulators (white bar and white hatched bar, respectively) or TALP without bicarbonate but supplemented with cAMP up-regulators (dark gray hatched bar) demonstrated a significant decrease in the percentage of non-responsive cells and increase in patterns indicative of cholesterol redistribution and efflux. Results are based on six independent samples and presented as the mean ± SEM. **P < 0.01 and ***P < 0.0001 indicate differences from the non-capacitating control.

Based on microscopic observations, there were four filipin-cholesterol complex patterns identified across the different conditions (Fig. 6A). Spermatozoa that showed a uniform distribution of filipin-cholesterol complexes across the entire head were identified as non-responding cells (Fig. 6A-1). In comparison, responsive cells were classified by the movement of filipin-cholesterol complexes to the apical and pre-equatorial regions of the sperm head with an exposure of the equatorial region (Fig. 6A-2 and 3). Under this classification, there were two different patterns of cholesterol redistribution, one with complete loss of fluorescence in the post-equatorial region (Fig. 6A-2) whereas in the other there was remnant fluorescence in this area of the sperm head (Fig. 6A-3). Spermatozoa with fewer filipin-cholesterol complexes across the whole sperm head (as identified by lower overall fluorescence), specifically when assessed against non-responsive or cholesterol redistributed patterns, were classified as cells that had undergone cholesterol efflux (Fig. 6A-4). With respect to the frequency of each of these patterns, irrespective of whether a condition was capacitating or non-capacitating, above 50% of spermatozoa were classified as non-responsive (Fig. 6B). When cells were exposed to TALP either with or without bicarbonate and cAMP up-regulators, there was a lower percentage of non-responsive cells and instead, a concomitant increase in patterns representing cholesterol redistribution or efflux when compared to the non-capacitating control. Since these conditions also were able to support extensive tyrosine phosphorylation (Supplementary Figs 2 and 3), this demonstrates that the redistribution of cholesterol and loss from the plasma membrane occurred as a result of capacitation. Out of all conditions that support capacitation, spermatozoa exposed to TALP devoid of bicarbonate with cAMP up-regulators presented the highest percentage of cells with cholesterol redistribution and efflux when compared to the non-capacitating control (Fig. 6B; 26.4 ± 2.5% and 11.3 ± 2.8%, respectively; P < 0.001).

## Discussion

The ability to reliably quantify cholesterol efflux is currently limited to methods that either require specialised equipment or that produce results that can be objectively difficult to interpret. This constraint in methodology not only hinders the attempt to study the role of cholesterol efflux during capacitation, but more importantly, it restricts the ability for researchers to better assess capacitation success *in vitro*. It was evident there was a need for a method to quantify cholesterol efflux that can be easily incorporated into sperm biology protocols, one that provides consistent results and utilises equipment that was more likely to be present in an andrology laboratory. In this manuscript we report the first documented use of the BODIPY-cholesterol assay in spermatozoa, which can successfully quantify cholesterol efflux during *in vitro* capacitation and has been validated with mass spectrometric analysis of endogenous cholesterol efflux. Prior to this, BODIPY-cholesterol had only been used in somatic cells as a means to study cholesterol efflux^28,29,31,32^. In these fixed cells lines, BODIPY-cholesterol was commonly measured by a fluorescent spectrophotometer following efflux from the cell to the supernatant, rather than measured directly from the cell itself as reported herein.

The loss of BODIPY-cholesterol differed significantly depending on what spermatozoa were exposed to in the media (Fig. 2A). This would indicate that this measured response is not random and is likely associated with a physiological change in these cells. In boar spermatozoa, the capacity to lose cholesterol is controlled by the degree of fluidity in the plasma membrane^7^, which is well known to be regulated by the cAMP-PKA pathway^6^. Though the predominant activator of this pathway is bicarbonate, this component can be readily replaced with chemicals that upregulate cAMP, such as cAMP analogues or phosphodiesterase inhibitors^6,33^. Furthermore, there is evidence that the presence of serum albumin to act as a cholesterol acceptor in boar spermatozoa is an absolute requirement as without it, cholesterol efflux cannot occur^7,18^. By examining the results of the current study more closely, in spermatozoa exposed to TALP supplemented with or without cAMP up-regulators or TALP devoid of bicarbonate with cAMP up-regulators, the loss of BODIPY-cholesterol is maximal when compared to the non-capacitating control (Fig. 2A). With this established knowledge of factors required for capacitation in boar spermatozoa, it confirms that the BODIPY-cholesterol assay is able to detect and quantify cholesterol efflux under conditions that support this process.

Whilst the evidence in literature suggests that cholesterol efflux measured with BODIPY-cholesterol is only occurring in certain conditions, these results required validation with a known gold standard, this being mass spectrometric (MS) analysis of endogenous cholesterol efflux. Using this method, it was found that only TALP with or without cAMP upregulation supported a significant 38–41% loss in endogenous cholesterol (Fig. 2B). Previous research investigating cholesterol efflux in boar spermatozoa verifies this finding, with up to 30% of endogenous cholesterol lost following exposure to TALP^7^. For boar spermatozoa, the action of bicarbonate or the replacement with cAMP up-regulators is sufficient to stimulate capacitation and support cholesterol efflux when in the presence of a cholesterol acceptor^7,33,34^. One might expect that if these two are combined together, that there may be an additive effect on the capacitation response owing to the upregulation of cAMP. In fact, it was observed that both TALP alone and in combination with cAMP up-regulators triggered a similar loss in cholesterol, which was not only quantified by MS analysis but also with the BODIPY-cholesterol assay (up to 33%). Moreover, it was found that under these cAMP upregulated conditions, sperm viability was significantly reduced (<50%). Collectively, these findings suggest that excessive stimulation of cAMP production in boar spermatozoa is superfluous and also highlights that even under extreme capacitation conditions, that there can be a limit to how much cholesterol can be lost from the membrane before it becomes detrimental to the cell^35^. The MS analysis also illustrated the parallelism between endogenous cholesterol efflux and BODIPY-cholesterol efflux, quantified with a flow cytometer (Fig. 2). Taken together, it is clear that the analysis of endogenous cholesterol loss validates the results of the BODIPY-cholesterol assay, verifying that this assay can reliably quantify cholesterol efflux from spermatozoa during *in vitro* capacitation.

A major benefit of the BODIPY-cholesterol assay is the ability to segregate populations based on viability using flow cytometry. To our knowledge, this technique has not yet been employed with somatic cells as most studies generally use fluorescent spectrophotometer to measure the loss of BODIPY-cholesterol in the surrounding medium^28,29,32,36^. As with many sperm function tests utilising flow cytometry, we were only concerned with isolating viable cells to measure the capacity for cholesterol efflux using BODIPY-cholesterol. Nonetheless, it became of interest to determine whether viability was an important factor to consider when quantifying cholesterol efflux either with this assay or MS analysis, particularly since the assessment of cholesterol efflux is mainly performed on the whole sperm population in the latter method. Based on the results, it was confirmed that after isolating the viable population, a similar loss in cholesterol was quantified with the BODIPY-cholesterol assay and MS analysis, which was in stark contrast to that observed in the non-viable population (Fig. 4). The reason behind this discrepancy between the two methods in the non-viable population is likely owing to additional cholesterol loss that is not attributed to the active process of cholesterol efflux. When capacitation in stimulated in spermatozoa, a proportion of these cells may become overstimulated and begin to deteriorate. Subsequently, the integrity of the plasma membrane declines, possibly leading to a loss of membrane from non-viable cells which could be detected as cholesterol efflux. This idea may also explain some of the differences observed between BODIPY-cholesterol and MS analysis in spermatozoa exposed to various conditions, since there was no segregation of viable sperm for this analysis (Fig. 2). In this respect, the BODIPY-cholesterol assay is more likely to better reflect which conditions were able to support the capacitation-related process of cholesterol efflux as opposed to the loss of plasma membrane. Whilst populations that are composed of a proportion of viable and non-viable cells appear to have a similar rate of cholesterol efflux, irrespective of the method used to quantify this loss, the fact that non-viable cells are included could reduce the meaningfulness of the results. This is not only an issue for MS analysis, the degradation of the plasma membrane may permit intracellular labelling with BODIPY-cholesterol that is not available for cholesterol efflux, thus causing an increase in the fluorescence measured in non-viable cells (Fig. 3C). When studying the importance cholesterol efflux during capacitation, the inclusion and examination of non-viable cells becomes irrelevant since these spermatozoa are unlikely to be involved in fertilisation. Based on this conclusion, when measuring cholesterol efflux with BODIPY-cholesterol or even with MS analysis, it is recommended that only the viable population is examined in order to produce biologically relevant results.

Labelling spermatozoa with filipin is another commonly used method of assessing cholesterol redistribution and efflux. This complex molecule is able to bind to endogenous cholesterol within cell membranes and it is intrinsically UV fluorescent, making it a useful tool for studying cholesterol changes in spermatozoa^37,38^. Whilst filipin labelling has been used in capacitation studies, there are pitfalls in attempting to quantify cholesterol efflux with this method. One major problem is that the rapid quenching of filipin can make visual analysis difficult and also it can be very subjective. Utilising flow cytometry for this purpose decreases subjectivity as all spermatozoa passing through the laser bundle will have the same exposure time to excitation light and will be enable to give exact comparable emission pulses that will be picked up by the detectors. Furthermore, due to the high throughput of thousands of cells in a short period of time, using flow cytometry increases the amount of data that would otherwise be collected by manual counting. It was surprising then that flow cytometry of filipin labelled boar spermatozoa showed no significant change in filipin fluorescence across any of the capacitating conditions when compared to the non-capacitated control (Fig. 5). Whilst there is evidence to suggest that differences in filipin fluorescence can be detected with flow cytometry after exposure to conditions that support cholesterol efflux (i.e. cAMP activation and presence of cholesterol acceptor)^17,25,26^, under our experimental conditions this was not observed. In one of the earliest studies using filipin to examine cholesterol changes in guinea pig spermatozoa, it was suggested that the frequency of filipin complexes is directly proportional to cholesterol residing in the plasma membrane^38^. More recently, the examination of freeze fracture scanning electron microscopy (SEM) images of filipin labelled boar spermatozoa suggested a different explanation of filipin-cholesterol binding. Through these images it was found that filipin formed intra-membrane complexes with cholesterol that could include multiple cholesterol molecules bound to a single filipin molecule^7^. In this respect, the amount of filipin fluorescence observed is not directly relative to the cholesterol content of the plasma membrane. This idea could explain the similar fluorescence detected from spermatozoa in this study regardless of whether the media could support capacitation or not. When the BODIPY-cholesterol assay was evaluated against filipin, it was clearly evident that BODIPY-cholesterol was able to not only detect cholesterol efflux using flow cytometry, but reliably quantify this loss (Fig. 2A). What we can take from this interpretation of the results is that filipin labelling is unlikely to be a dependable method for quantifying cholesterol efflux unlike BODIPY-cholesterol though it did prove useful for identifying changes in the distribution of cholesterol during capacitation.

In the current study, up to four filipin-cholesterol complex patterns were visualised with microscopy, these being a non-responsive pattern (Fig. 6A-1), responsive or cholesterol redistributed pattern (Fig. 6A-2 and 3) and a possible cholesterol loss pattern (Fig. 6A-4). Whilst two cholesterol redistribution patterns were identified, it is most likely that these patterns reflect different stages of cholesterol redistribution at the time of fixation and labelling. In this case, the classification of these patterns in this study was confirmed by a previous study in the boar which also identified these three main types of filipin-cholesterol complex patterns^7^. By further examining the frequency of these patterns, only when bicarbonate and/or cAMP up-regulators were present was there a significant increase in the percentage of cells demonstrating cholesterol redistribution (Fig. 6B). Moreover, if BSA was also supplemented to media, this resulted in a pattern that was indicative of cholesterol efflux. These responses are akin to what has been described in other studies utilising filipin to analyse cholesterol changes, both in the boar and other species like the stallion^7,24,33^. Interestingly, the conditions that supported enhanced cholesterol redistribution and apparent efflux as assessed with filipin corresponded with the same conditions that supported cholesterol efflux as measured by BODIPY-cholesterol (Fig. 2A). The similarities between these two methods reflect the efficacy of the BODIPY-cholesterol assay in detecting cholesterol efflux under conditions conducive for this process and further confirms the value of this assay for capacitation research.

Since capacitation is a fundamental maturation event for spermatozoa, any attempt to develop methodology to reliably measure its processes will be extremely useful for research focused on this area of sperm biology. In this manuscript we present the first validated flow-cytometry based assay to quantify cholesterol efflux in spermatozoa by utilising BODIPY-cholesterol. The potential for this assay extends beyond its use in boar spermatozoa to species where knowledge of the requirements or function of cholesterol efflux may be limited or even in a clinical setting where it may be used as a means to assess capacitation prior to fertilisation. The BODIPY-cholesterol assay provides andrology laboratories with an effective, reliable and simple method to detect and quantify cholesterol efflux, a hallmark of successful capacitation.

## Materials and Methods

### Chemicals

Unless otherwise stated, products were sourced from Sigma-Aldrich and were of the highest reagent grade available. BODIPY-cholesterol (TopFluor® Cholesterol; product No. 810255) and lipid standards were purchased from Avanti Polar Lipids (Ablaster, AL, USA). Propidium iodide was purchased from Invitrogen (Landsmeer, Netherlands) and Filipin complex (≥70% Filipin III) purchased from Sigma-Aldrich (Schnelldorf, Germany). All organic solvents used for lipid analysis were obtained from Labscan (Dublin, Ireland) and were of HPLC grade.

### Incubation media

The basal media used for this study was a modified Tyrodes medium supplemented with bovine serum albumin (BSA), lactate and pyruvate (TALP)^39^. TALP consisted of 2 mM CaCl_2_, 3 mM KCl, 0.4 mM MgCl_2_, 90 mM NaCl, 0.3 mM Na_2_HPO_4_, 10 mM HEPES, 21.6 mM Na lactate, 5 mM D-glucose, 2 mM Na pyruvate, 25 mM NaHCO_3_ and 3 mg/mL fatty acid free BSA. For the analysis of cholesterol efflux using any of the methods outlined in the objectives, spermatozoa were incubated in one of seven *in vitro* capacitation media conditions: (1) TALP alone, (2) TALP supplemented with 1 mM dibutyryl cAMP, caffeine and theophylline (cAMP up-regulators), (3) TALP devoid of fatty acid free BSA, (4) TALP devoid of fatty acid free BSA supplemented with cAMP up-regulators, (5) TALP devoid of bicarbonate, (6) TALP devoid of bicarbonate supplemented with cAMP up-regulators and (7) TALP devoid of bicarbonate and fatty acid free BSA (non-capacitating control). Capacitation media containing bicarbonate were equilibrated overnight with 5% CO_2_ in humidified atmosphere. If bicarbonate was removed from media, a molar equivalent of NaCl was added to ensure the osmolality was maintained. In the case of BSA removal, media was supplemented with a mixture of polyvinyl alcohol (PVA) and polyvinylpyrrolidone (PVP) (0.5 mg/mL of each). Where necessary, the pH of media was adjusted to 7.3 with NaOH and the osmotic pressure was measured as 300 ± 10 mOsm.

### Sperm preparation

The sperm-rich fractions of semen were collected from Large White boars (n = 12) with proven fertility kept at Varkens KI Nederland (Deventer, Netherlands), a facility that produces and distributes artificial insemination (AI) doses for sow herds commercially. One insemination dose from a boar contains approximately 1.5 × 10^7^ sperm in 80 mL with the semen diluted 1:10 (v/v) in Solusem® extender (Varkens KI Nederland, Deventer, Netherlands) for transport at 17 °C. Semen was processed for use no more than 24 h after delivery. Experimentation on these samples was approved by the ethics committee of Utrecht University and all experiments were performed in accordance with their guidelines and regulations. For mass spectrometric analysis (MS) and filipin labelling, spermatozoa from five or six boars were washed of seminal plasma and extender by centrifugation through a two-way step discontinuous gradient of isotonic Percoll® as described previously^40^. For *in vitro* capacitation, washed sperm pellets were diluted with each of the media conditions as outlined in *Incubation media* to a final concentration of 10 × 10^6^ sperm/mL (filipin labelling) or 20 × 10^6^ sperm/mL (MS analysis). Sperm suspensions containing bicarbonate were incubated in open tubes at 38.5 °C in a humidified cell incubator with 5% CO_2_ whereas non-bicarbonate containing samples were incubated in airtight tubes in heating block set to 38.5 °C for 2 h.

### BODIPY-cholesterol assay

To prepare a stock solution of BODIPY-cholesterol for this assay, the analogue was reconstituted in DMSO to a stock concentration of 1 mM and prior to storage, the solution was aliquoted into glass vials, flushed with N_2_ gas and stored at −80 °C until required. For the preparation of spermatozoa, cells diluted with BTS were washed (300 × g, 10 min) to remove excess diluent and seminal plasma and the loose pellet resuspended with non-capacitating control media to a concentration of 100 × 10^6^ sperm/mL. Sperm suspensions were labelled with BODIPY-cholesterol (final concentration of 0.4 µM), mixed thoroughly and incubated for 10 min at 37 °C. Excess BODIPY-cholesterol was removed via centrifugation through a two-way step discontinuous gradient of isotonic Percoll® (Fig. 1). The sperm pellet was diluted with each of the capacitation media conditions as outlined in *Incubation media* to a final concentration of 1 × 10^6^ sperm/mL and incubated for 2 h prior to flow cytometric assessment.

To discriminate between viable and non-viable cells in each sample, suspensions were incubated with propidium iodide (PI; 5 µg/mL) 10 min before the end of the incubation period. The analysis of BODIPY-cholesterol efflux and viability was performed on a BD FACSCANTO II flow cytometer following calibration (Becton Dickinson Biosciences). BODIPY-cholesterol and PI were excited by a 20 mW solid state laser with power at 488 nm. BODIPY-cholesterol fluorescence was detected on a 530/30 nm band-pass filter and PI fluorescence on 585/42 nm band-pass filter. A sperm cell specific population was gated based on forward light scatter and sideward light scatter profiles. For each sample, 10,000 events were recorded for further analysis in FlowJo (Version 10; FlowJo, LLC). No compensation was performed on the data collected. The BODIPY-cholesterol fluorescence was analysed in the gated viable (PI−) and non-viable (PI+) populations and the percent loss in fluorescence relative to the non-capacitating control was calculated for each sample (i.e. BODIPY-cholesterol fluorescence in TALP ÷ BODIPY-cholesterol fluorescence in control × 100; Fig. 1). To verify that cholesterol efflux quantified with the BODIPY-cholesterol assay was as a result of capacitation, tyrosine phosphorylation was assessed with immunostaining and western blotting (Supplementary Protocols S5 and 6).

In addition to flow cytometric assessment, both viable and non-viable BODIPY-cholesterol labelled spermatozoa were also microscopically examined using a fluorescent microscope (Leica SPE-II; DMI4000). Images were observed for regions and potential patterns of labelling that are the result of exposure to capacitating conditions.

### Lipid analysis of endogenous cholesterol efflux

Following *in vitro* capacitation, spermatozoa were washed by centrifugation (600 × g, 10 min) and the sperm pellet retained for lipid analysis. Lipids were extracted from spermatozoa according to Bligh and Dyer^41^ and dried under N_2_ gas. The dried lipids were resolubilised in a mixture of chloroform and methanol (1/1; v/v) containing sitosterol (20 μM) before injection into the multiple reaction monitoring LC/MSMS to analyse endogenous cholesterol. The lipids were separated on a Halo C18 fused core column (3.0 × 150 mm, 2.7 µm; Advanced Materials Tech, Wilmington, DE) using a gradient from 25% to 100% methanol/2-propanol (8/2; v/v) in methanol/water (1/1; v/v) in 1 min and an additional 13 min elution with methanol/2-propanol. Elution was at 40 °C and a flow rate of 300 µL/min. The effluent was introduced into a 4000 QTRAP MS instrument (Sciex, Concord, ON), fitted with an atmospheric pressure chemical ionization source operated at 400 °C and a needle current of 3 µA. Mass transitions were set to m/z ratio from precursor to most abundant fragment at optimal collision energy between 35–40 V^42^. The concentration of endogenous cholesterol in each sample was determined from the known cholesterol standard, sitosterol, and the percent loss in cholesterol compared to the non-capacitating control was calculated.

### Cholesterol efflux in sorted viable and non-viable sperm populations

Spermatozoa exposed to only TALP and non-capacitating control media for 2 h were firstly stained with PI (5 µg/mL) for 10 min before the end of the incubation period and then sorted with the BD Influx flow cytometer and cell sorter following calibration (see Supplementary Fig. S4; Becton Dickinson Biosciences). A sperm cell specific population was gated based on forward light scatter and sideward light scatter profiles and PI fluorescence was detected on a 585/42 nm band-pass filter. For each sort, 50,000 events were recorded for further analysis in FlowJo (Version 10; FlowJo, LLC). Cells were sorted based on two populations, viable (PI−) and non-viable (PI+), with 1 × 10^6^ cells collected and stored immediately at −20 °C for each sample. A sham sort was also performed to collect a combination of viable and non-viable cells (see Supplementary Fig. S4). After thawing, sperm pellets from each sample were collected by ultracentrifugation (126,000 × g, 70 min, 2 °C) to use for lipid analysis. Sperm pellets were processed and measured for endogenous cholesterol as described in *Lipid analysis of endogenous cholesterol efflux*. The percent loss in cholesterol compared to the non-capacitating control was calculated and these results were compared with that obtained in the BODIPY-cholesterol assay for all sperm populations.

### Detection of cholesterol redistribution and efflux using filipin

For filipin labelling, sperm suspensions were centrifuged to remove media after *in vitro* capacitation (600 × g, 10 min) and then immediately fixed for 30 min following resuspension with 4% paraformaldehyde (PFA) in PBS. Fixed cells were washed twice (600 × g, 5 min) in PBS and then resuspended with a PBS staining solution containing filipin complex dissolved in DMSO (≥70% Filipin III; final concentration of 83 µg/mL). Spermatozoa were labelled at room temperature for 1 h with gentle shaking and minimal light. Excess filipin was removed from spermatozoa by centrifugation (900 × g, 10 min) and cells were resuspended in PBS supplemented with 0.05% NaN_3_ for short term preservation. Samples were stored at 4 °C for no more than a week until required for microscopic analysis or flow cytometric assessment.

### Flow cytometric analysis of filipin fluorescence

Filipin labelled sperm were analysed with a BD Influx flow cytometer and cell sorter following calibration (Becton Dickinson Biosciences) to quantify changes in filipin fluorescence following *in vitro* capacitation. A sperm cell specific population was gated based on forward light scatter and sideward light scatter profiles. Filipin was excited by a UV laser (355 nm) and the fluorescence detected with a 550/40 nm band-pass filter. For each sample, 10,000 events were recorded for further analysis in FlowJo (Version 10; FlowJo, LLC). The percent loss in fluorescence compared to the non-capacitating control was calculated for each sample.

### Microscopic analysis of cholesterol redistribution

For the preparation of slides for microscopy, an aliquot of filipin labelled sperm suspension (approximately 50,000 cells) was placed on a coverslip and air dried. The prepared coverslips were mounted on a slide with Prolong Diamond Antifade Mountant (Invitrogen, Landsmeer, Netherlands) in order to reduce the rapid quenching of filipin fluorescence. All slides were microscopically examined for filipin-cholesterol complexes using a fluorescent microscope (Olympus BX60) equipped with an Hg lamp (120 W) and a UV filter block (360/20 nm band-pass excitation filter, 400 nm dichroic mirror and 425 nm long-pass emission filter). For all samples, at least 200 cells were counted across 4 fields and filipin-cholesterol patterns identified were represented as a percentage of the total number of cells.

### Statistical analysis

Cholesterol efflux as measured by the BODIPY-cholesterol assay (n = 6 ejaculates), mass spectrometric analysis (n = 5 ejaculates) and filipin (n = 6 ejaculates) was statistically analysed using linear mixed model regression (REML) in R 3.4.1. For the layout of these models, the media condition was set as a fixed effect and boar was included as a random effect. In the case of the comparison between the BODIPY-cholesterol assay and mass spectrometric analysis (for non-sorted and sorted cells), the method used to measure cholesterol efflux was also included as a fixed effect. The normality and homoscedasticity of the model residuals was checked with Shapiro-Wilk test and Bartlett’s test, respectively. Pairwise comparisons were performed using Tukey’s adjustment and the effect of heteroscedasticity in the residuals was reduced when necessary. This was achieved by separating the variance of the fixed effect according to its levels using the function ‘varIdent’ before performing traditional REML. Data are presented as the mean ± S.E.M. and results that are P < 0.05 are considered significant.

## Supplementary information

Supplementary information

## Data Availability

The datasets generated during and/or analysed during the current study are available from the corresponding author upon request.
