# Supplementary material for: BODIPY-cholesterol can be reliably used to monitor cholesterol efflux from capacitating mammalian spermatozoa
Source: Sci Rep. 2019 Jul 8;9:9804. doi: 10.1038/s41598-019-45831-7 (PMC6614389; doi:10.1038/s41598-019-45831-7)
Supplement: Supplementary file 1 — Supplementary information [file 41598_2019_45831_MOESM1_ESM.pdf]

Supplementary Information

BODIPY-cholesterol can be reliably used to monitor cholesterol efflux from capacitating mammalian spermatozoa

AUTHOR LIST AND AFFILIATIONS
N.C. Bernecic*[1], M. Zhang[2], B.M. Gadella[2,3], J.F.H.M. Brouwers[2], J.W.A. Jansen[2], G.J.A. Arkesteijn[2], S.P. de Graaf[1] and T. Leahy[1]
[1] The University of Sydney, Faculty of Science, NSW 2006, Australia
[2] Department of Biochemistry & Cell Biology, Utrecht University, The Netherlands
[3] Department of Farm Animal Health, Faculty of Veterinary Medicine, Utrecht University, The Netherlands

*Email: naomi.bernecic@sydney.edu.au

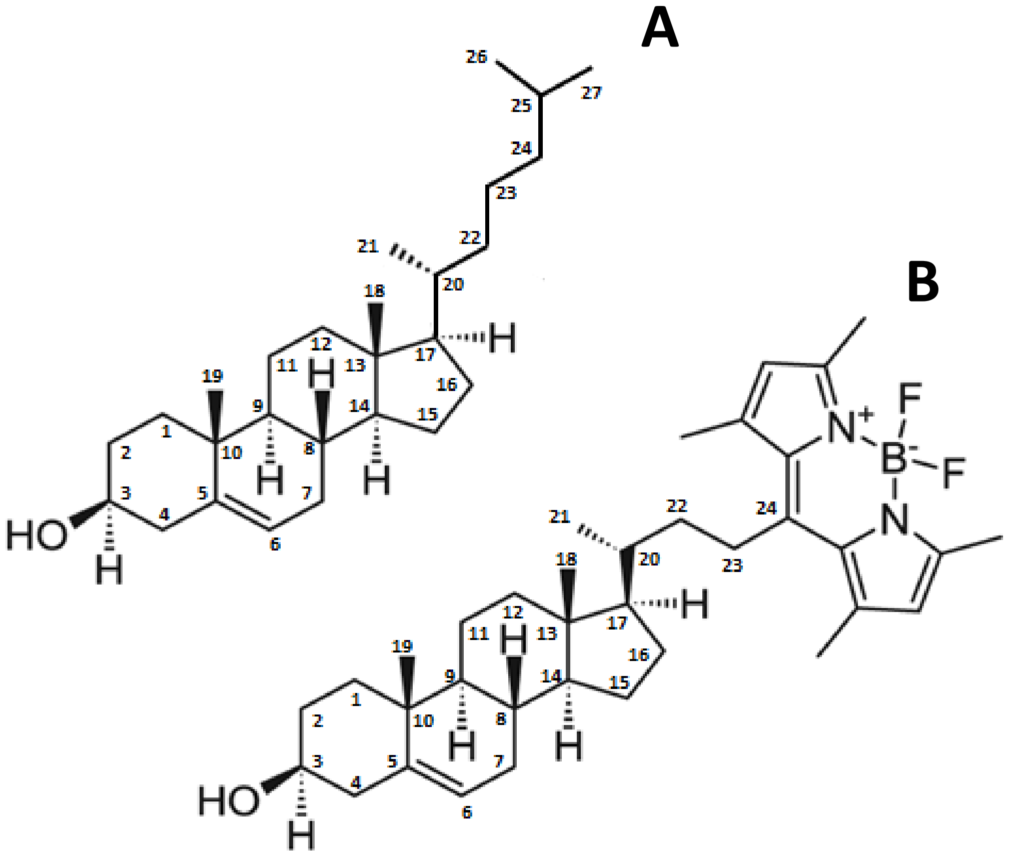

Supplementary Figure S1. A native cholesterol molecule (**A**) compared with the BODIPY-cholesterol fluorophore used to quantify cholesterol efflux in spermatozoa (**B**). Both molecules share the four hydrocarbon rings that form the bulky part of the structure though in the case of BODIPY-cholesterol, part of the hydrophobic tail is replaced by the fluorescent boron dipyrromethene difluoride (BODIPY) tag which is conjugated to the 24-carbon. Images adapted from Avanti Polar Lipids, Inc.

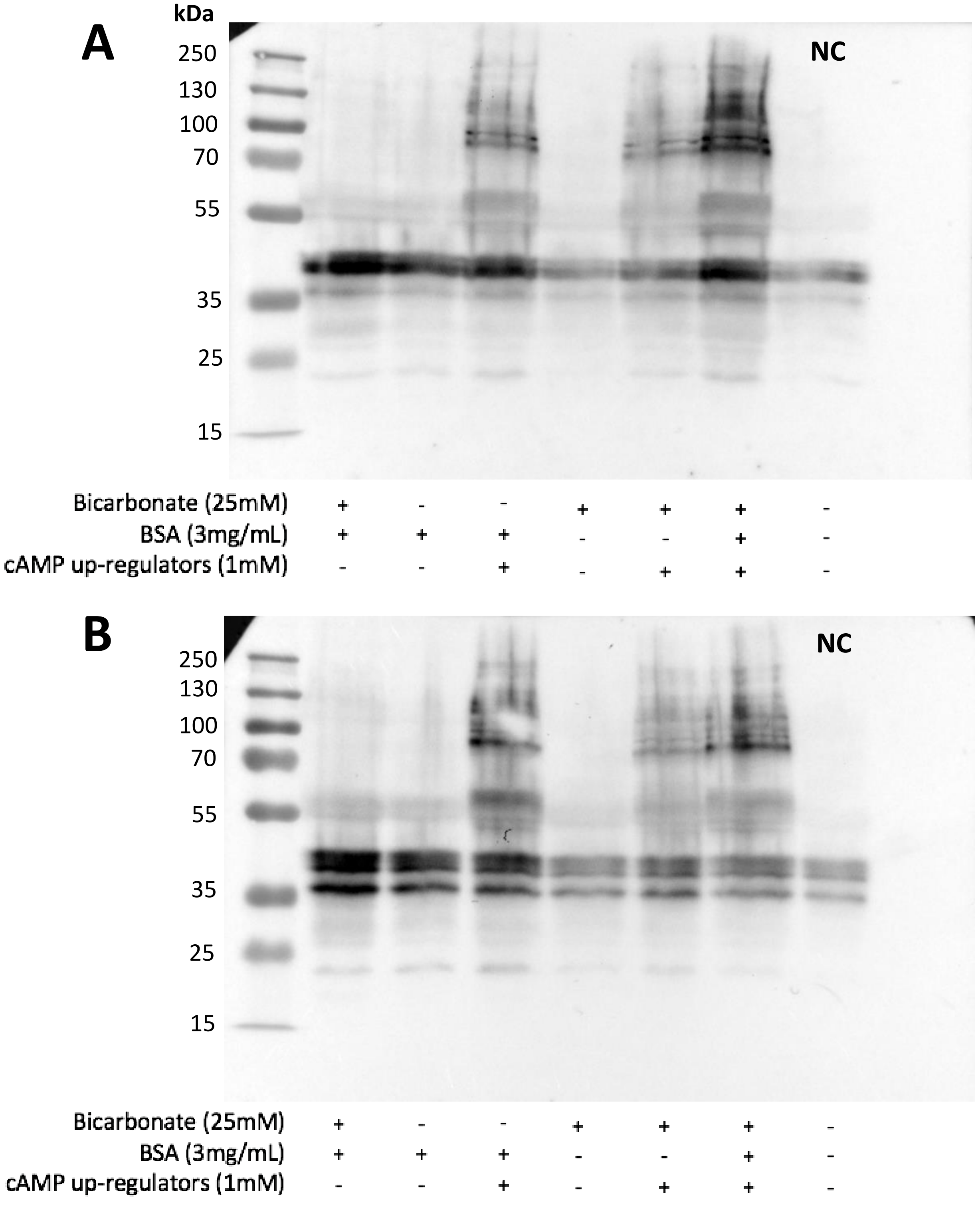

Supplementary Figure S2. Assessment of tyrosine phosphorylation of boar sperm proteins following exposure to capacitating and non-capacitating conditions for 2 h. In contrast to the non-capacitating control (**NC**), tyrosine phosphorylation was present in all conditions supplemented with bicarbonate and BSA or when these components were replaced with cAMP up-regulators, indicating that capacitation was supported. Representative blots are shown for two boars (**A** and **B**) and were taken at a single exposure.

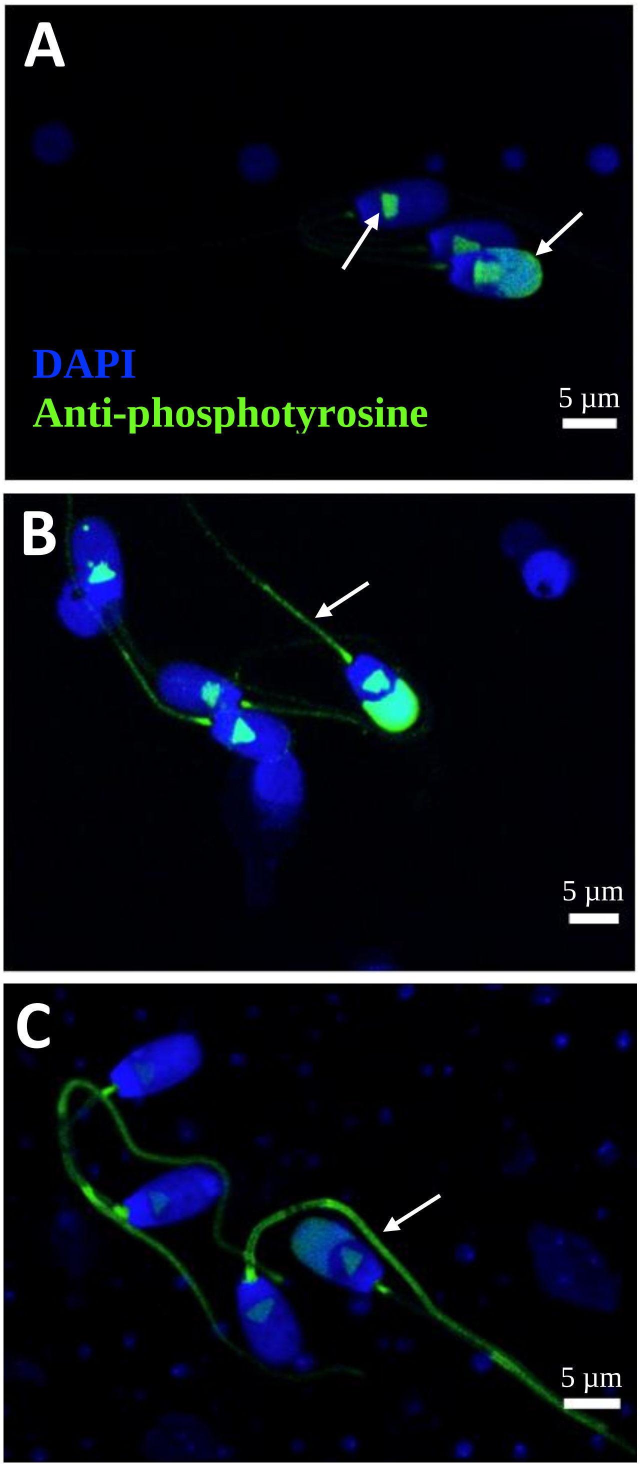

Supplementary Figure S3. Immunolocalisation of tyrosine phosphorylation on boar spermatozoa exposed to capacitating and non-capacitating conditions for 2 h. In all conditions, tyrosine phosphorylation was present in the equatorial and/or apical region of the sperm head (as indicated by arrows in the non-capacitating control; **A**). Only in conditions that supported capacitation, such as TALP (**B**), was there evidence of tyrosine phosphorylation in the mid piece of the tail. The supplementation of cAMP up-regulators in TALP without bicarbonate (**C**) was able to support extensive tyrosine phosphorylation in the mid and/or principle piece of the tail. These results indicate the ability for cAMP up-regulators to readily replace bicarbonate and stimulate capacitation, as also observed in the Western blots. Scale bar = 5μm.

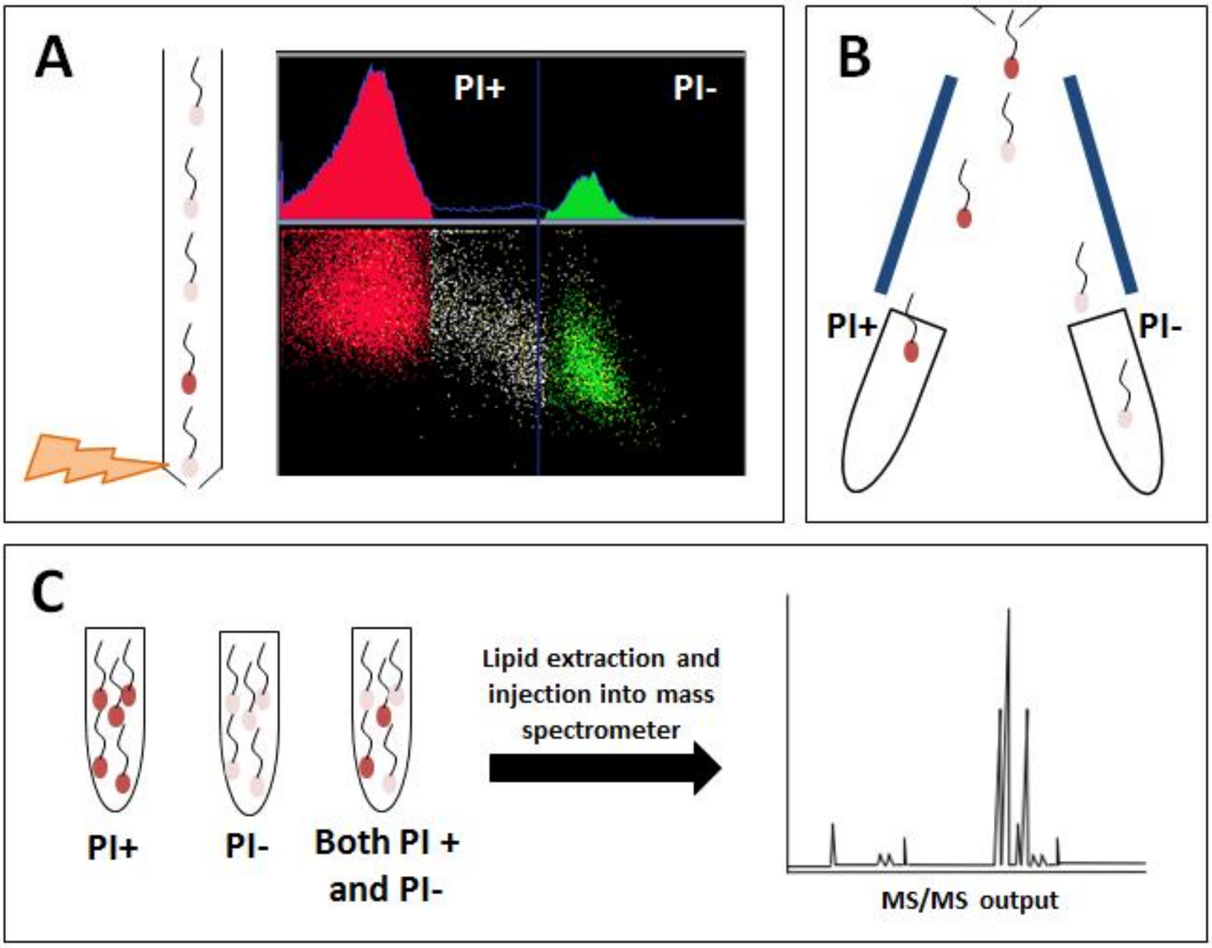

Supplementary Figure S4. Analysis of endogenous cholesterol efflux from boar spermatozoa segregated into populations based on viability. (**A**) Boar spermatozoa were first stained with propidium iodide (PI) and analysed with flow cytometry to detect viable (PI-) and non-viable (PI+) sperm populations. (**B**) Spermatozoa were sorted based on viability into separate tubes and a sham sort was performed to collect a sample with a mixture of viable and non-viable cells. (**C**) Spermatozoa across all populations were pelleted by ultracentrifugation and sperm lipids were extracted from the resulting pellet. Sitosterol was included in the lipid extraction as a reference for extraction efficiency and as a known standard to determine the cholesterol concentration in each sample. Extracted lipids were injected directly into the LC MS/MS for analysis of cholesterol efflux.

Supplementary Protocol S5

Indirect immunofluorescent staining

Following incubation in various conditions for 2 h, boar spermatozoa were fixed with 4% paraformaldehyde (PFA; w/v) for 30 minutes at room temperature and air dried on slides. Spermatozoa were then permeabilized using 0.5% Triton X-100 (v/v) for 15 min at room temperature. After rising with PBS, slides were incubated with 1% BSA in PBS (w/v; PBS-BSA) for 1 h at room temperature and incubated overnight at 4°C with mouse anti-phosphotyrosine antibody (1:100 dilution; clone 4G10®; Merck Millipore, Darmstadt, Germany) in PBS-BSA. Prepared slides were washed three times with PBS-BSA and incubated for 1 h at room temperature with Alexa Fluor 488-conjugated goat anti-mouse IgG (1:100 dilution; Thermo Fisher Scientific, Breda, the Netherlands). Spermatozoa were counterstained with 10 µM Hoechst 33342 (Sigma, St. Louis, MO, USA) in order to identify nuclei. After extensive washing with PBS, slides were mounted with FluorSave™ reagent (Calbiochem, San Diego, CA, USA) to minimize quenching and covered with a coverslip. All slides were microscopically examined using a Leica TCS SP2 confocal system (Leica Microsystems GmbH, Wetzlar, Germany). Alexa Fluor 488 and Hoechst 33342 were excited using a 488 nm or 405 nm laser and the fluorescence detected using 480±10 nm or 528±10 nm band-pass filter, respectively. Images were evaluated for the presence of tyrosine phosphorylation and the extent of fluorescence across different regions of the cell. Representative images of boar spermatozoa with tyrosine phosphorylation are presented in Supplementary Figure S2.

Supplementary Protocol S6

Western blotting

An aliquot of 10 x 10^6 spermatozoa was lysed in 4X sample buffer (50 mM Tris, pH 6.8, 2.5% β-mercapto-ethanol (w/v), 2% SDS, 0.02% bromophenol blue (w/v), 10% glycerol (w/v)) at room temperature for 30 min and vortexed every 10 min. Lysed samples were then diluted 1:3 with MilliQ water and boiled for 10 minutes. Cell debris was removed by centrifugation at 14,000 x g for 20 min. The molecular weight marker (PageRuler™ Plus Pre-stained Protein Ladder; Thermofisher; Waltham, MA) and samples were loaded into each lane of a gel and subjected to SDS-PAGE with a 4% stacking gel and 12% running gel. Gels were run for 20 min at 100V then a further 60 min at 120V. Proteins were transferred onto polyvinylidene difluoride (PVDF) membrane (GE Healthcare; Eindhoven, The Netherlands) for 60 min at 100V. Membranes were blocked in 5% BSA (w/v) in PBS with 0.05% Tween-20 (v/v; PBS-T) for 3 h at room temperature in. Following blocking, membranes were incubated with the mouse anti-phosphotyrosine antibody in PBS-T with 1% BSA (w/v) (diluted 1:2000; clone 4G10®; Merck Millipore, Darmstadt, Germany) overnight at 4°C. The membrane was then washed three times for 15 min in PBST and incubated with the secondary antibody, goat anti-mouse HRP IgG in PBS-T with 1% BSA (w/v) (diluted 1:10000, Nordic-MUbio, Tilburg, The Netherlands) for 1 h at room temperature. After washing four times for 20 min in PBS-T, the membranes were developed using chemiluminescence (ECL-detection kit; Supersignal West Pico, Pierce, Rockford, IL, USA) and visualized with a ChemiDoc (Bio-Rad).
